# Inhibition of upper small intestinal mTOR lowers plasma glucose levels by inhibiting glucose production

**DOI:** 10.1038/s41467-019-08582-7

**Published:** 2019-02-12

**Authors:** T. M. Zaved Waise, Mozhgan Rasti, Frank A. Duca, Song-Yang Zhang, Paige V. Bauer, Christopher J. Rhodes, Tony K. T. Lam

**Affiliations:** 1 Toronto General Hospital Research Institute, UHN, Toronto, ON M5G 1L7 Canada; 2 Department of Physiology, University of Toronto, Toronto, ON M5S 1A8 Canada; 3 Kovler Diabetes Center, Department of Medicine, Section of Endocrinology, Diabetes and Metabolism, University of Chicago, Chicago, IL 60637 USA; 4 Department of Medicine, University of Toronto, Toronto, ON M5S 1A8 Canada; 5 Banting and Best Diabetes Centre, University of Toronto, Toronto, ON M5G 2C4 Canada; 6 Present Address: School of Animal and Comparative Biomedical Sciences, University of Arizona, Tucson, AZ 85721 USA; 7 Present Address: MedImmune LLC, Gaithersburg, MD 20878 USA

## Abstract

Glucose homeostasis is partly controlled by the energy sensor mechanistic target of rapamycin (mTOR) in the muscle and liver. However, whether mTOR in the small intestine affects glucose homeostasis in vivo remains unknown. Here, we first report that delivery of rapamycin or an adenovirus encoding the dominant negative acting mTOR-mutated protein into the upper small intestine is sufficient to inhibit small intestinal mTOR signaling and lower glucose production in rodents with high fat diet-induced insulin resistance. Second, we found that molecular activation of small intestinal mTOR blunts the glucose-lowering effect of the oral anti-diabetic agent metformin, while inhibiting small intestinal mTOR alone lowers plasma glucose levels by inhibiting glucose production in rodents with diabetes as well. Thus, these findings illustrate that inhibiting upper small intestinal mTOR is sufficient and necessary to lower glucose production and enhance glucose homeostasis, and thereby unveil a previously unappreciated glucose-lowering effect of small intestinal mTOR.

## Abstract (web summary)

The mechanistic target of rapamycin (TOR) functions as an energy sensor and contributes to the control of glucose homeostasis. Here, the authors show that mTOR in the upper small intestine regulates hepatic glucose production and is required for the glucose lowering effect of metformin.

## Introduction

The mechanistic target of rapamycin (TOR), a highly conserved serine–threonine kinase, was first discovered in yeast in 1991^1^ and reported to act as an energy sensor in mammals^2^. This mammalian homolog (mTOR) is known to integrate input from nutrients (i.e., glucose, amino acids, and fatty acids), hormones (i.e., insulin), and cytokines to regulate cell growth, metabolism, and development^3–5^. Dysregulation of mTOR is implicated in a number of human pathologies, including diabetes and obesity^6–8^. In fact, mTOR inhibitors are potential drugs primarily tested to treat cancer and metabolic diseases by altering cell growth and lipid and glucose metabolism^7, 9^. Consistent with the development of mTOR inhibitors as a glucose-lowering agent, mTOR is over-activated in high-fat diet (HFD)-induced obese rodents and induces insulin resistance in the liver and muscle^10, 11^; whereas the downregulation of mTOR signaling, or the deletion of S6 kinase (S6K), the crucial effector of mTOR signaling, is sufficient to enhance insulin sensitivity in rodents^12, 13^. Moreover, a single oral dose of rapamycin, a macrolide originally isolated from the soil bacterium *Streptomyces hygroscopicus*, inhibits mTOR and improves insulin sensitivity in humans^14^. Collectively, these findings implicate mTOR in the control of glucose homeostasis.

**Fig. 1:**
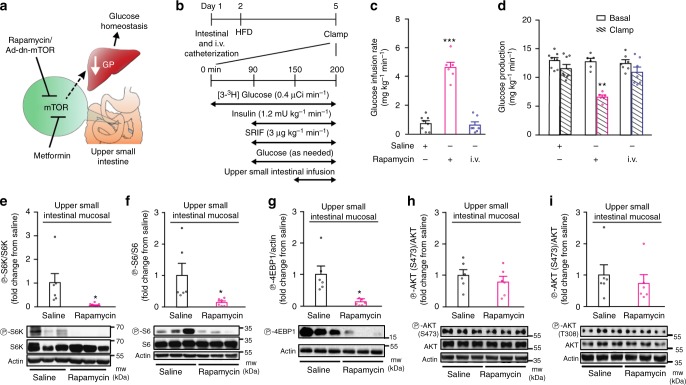
Upper small intestinal rapamycin infusion lowers glucose production and inhibits mTOR signaling. **a** Schematic representation of the working hypothesis. **b** Experimental procedure and pancreatic (basal insulin)–euglycemic clamp protocol. SRIF, somatostatin; µCi, microcurie. **c**, **d** The glucose infusion rate (**c**) and glucose production (**d**) during the clamp in HFD rats infused with upper small intestinal saline (*n* *=* 8) or rapamycin (*n* *=* 6) or with intravenous (i.v.) rapamycin (*n* *=* 7). **p *<* 0.01 or ***p *<* 0.001 vs. all other groups as determined by ANOVA with Tukey’s post hoc test. **e**–**i** Quantitative analysis and representative western blot of phosphorylated S6K (**e**), S6 (**f**), 4EBP1 (**g**), AKT (S473) (**h**), and AKT (T308) (**i**) protein expression in the upper small intestinal mucosa of HFD rats infused for 50 min with upper intestinal saline or rapamycin. The phosphorylation level was quantified by densitometry and data are presented as fold increase in rapamycin (*n* *=* 6) over the saline (*n* *=* 6) treated samples. Actin, loading control. *p *<* 0.05 vs. saline as calculated by unpaired *t*-test. Values are shown as mean ± s.e.m.

In parallel, nutrient-sensing signals in the small intestine play a significant role in the regulation of glucose homeostasis by influencing hepatic glucose production and/or overall blood glucose levels^15–18^. For example, the antidiabetic clinical drug metformin or the glucose-lowering agent resveratrol activates upper small intestinal energy sensor protein AMP-kinase (AMPK) to lower hepatic glucose produciton and restore glucose homeostasis in diabetic rodents^19, 20^. Although the intestinal mechanism of metformin action remains largely unknown, small intestinal-restricted exposure of metformin lowers glucose in people with type 2 diabetes^21^, while metformin inhibits mTOR signaling by both AMPK dependent and independent pathways^22–24^. Based on these complementary findings, we tested the working hypothesis that upper small intestinal mTOR-dependent pathways play a role in glucose homeostasis in vivo and is required for the glucose-lowering effect of metformin (Fig. 1a). Here, we report that inhibition of upper small intestinal mTOR signaling is necessary and sufficient to improve glucose homeostasis by lowering glucose production in rodents in vivo.

## Results

### Upper small intestinal mTOR regulates glucose production

To begin testing whether upper small intestinal mTOR inhibition regulates glucose homeostasis, we infused rapamycin or saline (control) directly into the lumen of the upper small intestine in rats that had comparable body weights (Supplementary Figure 1a) and fed a HFD for 3 days. The HFD rats were hyperphagic (Supplementary Fig. 1b) and hyperinsulinemic (Supplementary Fig. 1c) (an indication of insulin resistance as previously assessed^20^). We first performed pancreatic (basal insulin)-euglycemic clamps combined with tracer glucose infusion to assess steady-state changes in glucose metabolism independent of changes to glucoregulatory hormones in response to upper small intestinal infusion (Fig. 1b). When rapamycin was infused over the course of 50 min (1 µg/min; 50 µg; ~165 µg/kg) into the upper small intestinal lumen of HFD rats, plasma glucose levels were lowered such that the glucose infusion rate required to maintain euglycemia was increased compared to saline control (Fig. 1c and Supplementary Table 1). This increase in glucose infusion rate was due to a decrease in glucose production (Fig. 1d and Supplementary Fig. 1d) as the rate of glucose uptake remained comparable (Supplementary Fig. 1e). This effect of rapamycin vs. saline was accompanied by a downregulation of upper small intestinal mTOR signaling as indicated by a decrease in the phosphorylation of ribosomal S6 kinase (S6K) (Fig. 1e), S6 ribosomal protein (S6) (Fig. 1f), and eukaryotic translation initiation factor 4E binding protein 1 (4EBP1) (Fig. 1g) in the upper small intestinal mucosal tissues taken immediately after the infusion-clamp studies. Importantly, inhibition of mTOR signaling was restricted to the upper small intestine as the phosphorylation of S6K and S6 in the livers were not affected by upper small intestinal rapamycin infusion (Supplementary Fig. 1g and h), indicating that the rapamycin’s effects derived from the currently low-dose rapamycin 50-min infused protocol remains preabsorptive.

In the current experimental condition while plasma insulin levels were maintained at basal levels during the pancreatic-euglycemic clamps (i.e., a non-insulin-stimulated condition) (Supplementary Table 1), we found that upper small intestinal infusion of rapamycin for 50 min did not alter upper small intestinal AKT (residue S473 and T308) phosphorylation (Fig. 1h, i) in HFD rats, consistent with the fact that an acute (1 h) rapamycin treatment did not alter AKT phosphorylation (both at S473 and T308 residues) in non-insulin-stimulated in vitro conditions^25, 26^. We neither detected any changes in upper small intestinal gluconeogenic gene expression after 50 min of upper small intestinal rapamycin infusion as well (Supplementary Fig. 1f). In addition, when rapamycin was infused intravenously at a comparable dose and duration as the upper small intestinal rapamycin infusion, rapamycin failed to alter glucose homeostasis (Fig. 1c, d and Supplementary Fig. 1d, e) but inhibited hepatic mTOR signaling (Supplementary Fig. 1g and h). This is consistent with the fact that although hepatic-specific knockdown of S6K enhances the ability of circulating hyperinsulinemia to lower glucose production in HFD rodents, basal glucose production during non-insulin-stimulated conditions did not alter in the HFD-hepatic-specific S6K knockdown vs. HFD-control rodents^27^. Overall, these findings indicate that upper intestinal rapamycin infusion preabsorptively inhibits upper small intestinal mTOR signaling and lowers glucose production in HFD rats.

**Fig. 2:**
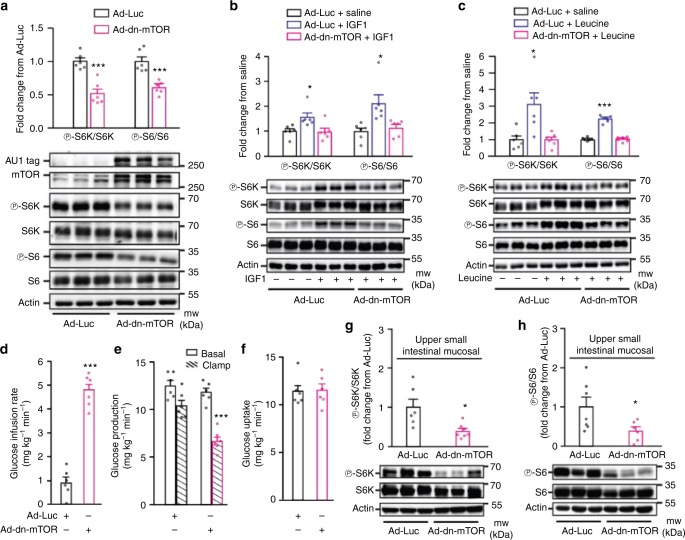
Molecular inhibition of upper small intestinal mTOR lowers glucose production. **a**–**c** Representative western blot and quantitative analysis of phosphorylated S6K and S6 protein expression normalized to their respective total in PC12 cells infected with Ad-dn-mTOR and treated with saline (**a**), IGF1 (**b**), or leucine (**c**) compare to cells infected with Ad-Luc and treated with saline. AU1, tag protein for mTOR construct; actin, loading control. **a** ***p < 0.001 as calculated by unpaired *t*-test (*n* = 6 for each group); **b**, **c** *p < 0.05 or ***p < 0.001 vs. all other groups as determined by ANOVA with Tukey’s post hoc test (*n* = 6 for each group). **d**–**f** The glucose infusion rate (**d**), glucose production (**e**), and glucose uptake (**f**) during the clamp in HFD-fed rats with upper small intestinal Ad-Luc (*n* = 6) or Ad-dn-mTOR (*n* = 6). ***p *<* 0.001 vs. Ad-Luc as calculated by unpaired *t*-test. **g**, **h** Quantitative analysis and representative western blot of phosphorylated S6K (**g**) and S6 (**h**) protein expression normalized to their respective total in the upper small intestinal mucosal tissue of HFD rats with either Ad-Luc (*n* = 6 for S6k, and 7 for S6) or Ad-dn-mTOR (*n* = 7 for S6k, and 6 for S6). *p < 0.05 vs. Ad**-**Luc as calculated by unpaired *t*-test. Actin, loading control. Values are shown as mean ± s.e.m.

We alternatively evaluated the effect of upper small intestinal mTOR inhibition on glucose homeostasis via the use of an adenovirus (Ad) expressing the dominant negative (dn) form of mTOR (Ad-dn-mTOR) (Fig. 1a). The Ad-dn-mTOR variant carries an Asp2338 to Ala mutation that rendered it catalytically inactive (loss-of-function)^28, 29^. Of note, a Ad-ca-mTOR variant was generated in parallel as well by deleting amino acid sequences 2430–2450 with fivefold higher activity than that of wild type mTOR (see below gain-of-function studies)^28, 29^. The viral-mediated expression of the mTOR mutants would compete with the endogenous mTOR and alter the overall activity of mTOR. We here first confirmed that Ad-dn-mTOR vs. control (Ad-Luc) constructs increased AU1 tag protein and mTOR expression (Fig. 2a). Ad-dn-mTOR vs. Ad-Luc decreased phosphorylated S6K and S6 proteins (Fig. 2a), and negated the stimulatory effect of both insulin-like growth factor 1 and leucine on mTOR signaling (Fig. 2b, c).

Next, we used an upper small intestinal-targeted viral approach^30^ where we injected either Ad-dn-mTOR or Ad-Luc into the upper small intestine 3 days prior to the infusion-clamp experiments to inhibit upper small intestinal mTOR, and on the day of the clamps, Ad-dn-mTOR vs. Ad-Luc did not affect body weight (Supplementary Fig. 2a). During the pancreatic clamps, the Ad-dn-mTOR infected rats exhibited a higher glucose infusion rate (Fig. 2d) and lower glucose production (Fig. 2e and Supplementary Fig. 2b) without any alteration in glucose uptake (Fig. 2f) as compared to Ad-Luc rats. Upper small intestinal mTOR signaling in rats with Ad-dn-mTOR transduction was inhibited by ~50% as confirmed by a reduction in S6K and S6 phosphorylation (Fig. 2g, h), and this mTOR inhibition had no effect on AKT (residue S473 and T308) phosphorylation (Supplementary Fig. 2c and d) in the current experimental pancreatic (basal insulin)-euglycemic context. Infusing rapamycin into the upper small intestine of Ad-dn-mTOR rats that would have further inhibited mTOR signaling to ~ 90% (Fig. 1e, f) did not additionally alter glucose metabolism (Supplementary Fig. 2e-g) as compared to rapamycin or Ad-dn-mTOR alone, suggesting that a threshold of ~ 50% inhibition on small intestinal mTOR is sufficient to exert a substantial effect on glucose production in rodents in vivo.

Considered altogether with the rapamycin-targeted loss-of-function studies, our experiments indicate that inhibition of upper small intestinal mTOR is sufficient to lower glucose production in HFD rodents.

### Upper small intestinal mTOR is required for metformin action

**Fig. 3:**
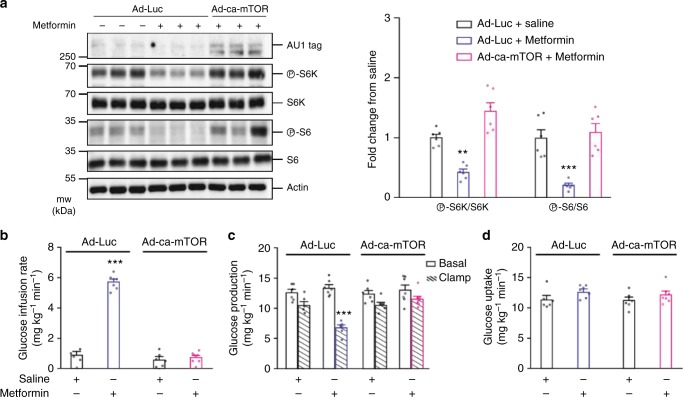
Upper small intestinal mTOR inhibition is necessary for metformin action. **a** Representative western blot (left) and quantitative analysis (right) of phosphorylated S6K, and S6 protein expression normalized to their respective total protein expression in HEK293 cells infected with either Ad-Luc or Ad-ca-mTOR and then treated with saline or metformin for 12–16 h. AU1, tag protein for mTOR construct; Actin, loading control. **p *<* 0.01 or ***p *<* 0.001 vs. all other groups as determined by ANOVA with Tukey’s post hoc test (*n* = 6 for each group). **b**–**d** Glucose infusion rate (**b**), glucose production (**c**), and glucose uptake (**d**) during the clamp in HFD-fed rats infected with either upper small intestinal Ad-Luc or Ad-ca-mTOR and infused with upper intestinal saline (*n* = 6 for each group) or metformin (*n* *=* 7 for each group). ***p *<* 0.001 vs. all other groups as determined by ANOVA with Tukey’s post hoc test. Values are shown as mean ± s.e.m.

To test for a potential necessity of mTOR function in the glucose-lowering effect of metformin treatment, we first demonstrated that incubation of Ad-Luc (control) viral-infected HEK293 cells with metformin resulted in a lower ratio of phosphorylated S6K and S6 to their respective total than those treated with saline (Fig. 3a). However, when the cells were instead infected with the adenovirus encoding the constitutively acting mTOR-mutated protein (Ad-ca-mTOR; see description above) the inhibitory effects of metformin on mTOR signaling were negated (Fig. 3a), confirming the functional role of Ad-ca-mTOR. We next performed an upper small intestinal-targeted viral injection of Ad-ca-mTOR or Ad-Luc into HFD rats 3 days prior to the infusion-clamp studies aimed to negate the inhibitory effect of metformin on mTOR and evaluate whether glucose control is subsequently lost in vivo. On the day of the clamps, we first noted that Ad-ca-mTOR vs Ad-Luc did not affect body weight (Supplementary Fig. 3a) but increased upper small intestinal pS6K and pS6 (Supplementary Fig. 3c and d) without affecting pAKT S473 (Supplementary Fig. 3e). We next confirmed that a 50 min upper small intestinal infusion of metformin at 200 mg kg^−1^ (which acts via preabsorptive mechanisms as demonstrated^19^) increased the glucose infusion rate (Fig. 3b) and lowered glucose production (Fig. 3c and Supplementary Fig. 3b) compared to saline control without affecting glucose uptake (Fig. 3d) in Ad-Luc rats. We then found that rats injected with Ad-ca-mTOR failed to respond to the metformin infusion and exhibited glucose kinetics similar to rats that received saline infusion (Fig. 3b–d and Supplementary Fig. 3b). Collectively, this indicates that metformin acts via a preabsorptive mTOR inhibitory-dependent mechanism in the upper small intestine to lower glucose production in HFD rodents.

### mTOR lowers hepatic glucose production via a gut–brain axis

**Fig. 4:**
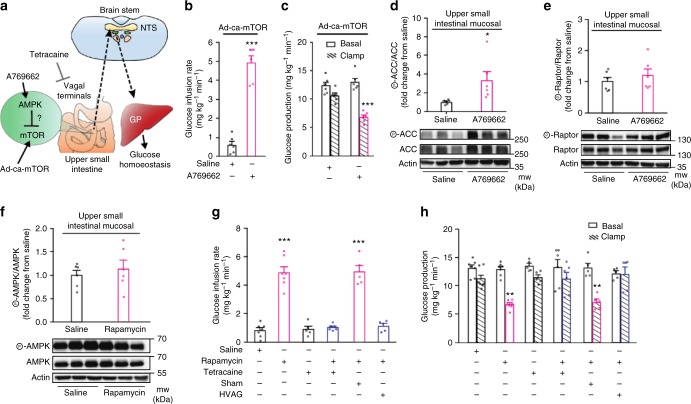
Inhibition of upper small intestinal mTOR activates a gut–brain axis to lower hepatic glucose production. **a** Schematic representation of working hypothesis. **b**, **c** Glucose infusion rate (**b**) and glucose production (**c**) during the clamps in HFD rats infected with upper small intestinal Ad-ca-mTOR and infused with upper intestinal A769662 or saline. ***p *<* 0.001 vs. saline; determined by unpaired *t*-test (*n* = 6 for each group). **d**, **e** Quantitative analysis and representative western blot of phosphorylated ACC (**d**) and raptor (**e**) protein expression in the upper intestinal mucosa of Ad-ca-mTOR infected HFD rats infused with upper intestinal saline or A769662. Actin, loading control. *p *<* 0.05 vs. saline as calculated by unpaired *t*-test (*n* = 5 for saline, and 6 for A769662 in ACC, and 6/group in raptor). **f** Western blot of phosphorylated AMPK protein expression in the upper small intestinal mucosa of HFD rats infused with upper intestinal saline or rapamycin. Actin, loading control. *p *<* 0.05 vs. saline as calculated by unpaired *t*-test (*n* = 6 for each group). **g**, **h** Glucose infusion rate (**g**) and glucose production (**h**) during clamps in HFD rats infused with upper small intestinal saline (*n* = 7), rapamycin (*n* = 7), tetracaine (*n* = 5), rapamycin + tetracaine (*n* = 6), rapamycin + sham (*n* *=* 5) or hepatic vagal branch vagotomy (HVAG) (*n* *=* 5). **p < 0.01 or ***p < 0.001 vs. saline, tetracaine, tetracaine + rapamycin, and HVAG + rapamycin; determined by ANOVA with Tukey’s post hoc test. Values are shown as mean ± s.e.m.

Given that metformin inhibits mTOR signaling by both AMPK dependent and independent pathways^22–24^ and that direct AMPK activation inhibits mTOR^31, 32^, we next evaluated whether the previously described metformin-AMPK axis in the upper small intestine that lower glucose levels^19^ is dependent on mTOR inhibition in vivo. We directly activated upper small intestinal AMPK via AMPK activator A769662 infusion administered at a dose that has been previously documented to mimic the glucose-lowering effect of upper small intestinal metformin infusion^19^. In the presence of Ad-ca-mTOR transduction in HFD rats (Fig. 4a), we found that upper small intestinal infusion of A769662 is equally effective and sufficient to increase glucose infusion rate and lower glucose production independent of changes in glucose uptake (Fig. 4b, c and Supplementary Fig. 4a, b). Consistently, A769662 increased p-ACC/ACC in upper small intestinal mucosal tissues obtained immediately after the clamp studies (Fig. 4d), but failed to alter p-raptor (Fig. 4e), probably due to the fact that ACC vs. raptor is a higher affinity substrate of AMPK^33–35^. Conversely, upper small intestinal infusion of rapamycin that inhibited mTOR signaling (Fig. 1e–g) did not alter p-AMPK (Fig. 4f). Thus, our findings indicate that upper small intestinal mTOR inhibition is not necessary for AMPK activtion to lower glucose production in HFD rodents.

As a gut–brain axis is necessary for the preabsorptive effect of upper small intestinal metformin to lower glucose production^19^, we next investigated whether a vagal neuronal innervation of the upper small intestinal as well as the hepatic vagus is necessary for the glucose production-lowering effect of upper small intestinal mTOR inhibition (Fig. 4a). We first coinfused the topical anesthetic tetracaine with rapamycin into the upper small intestine of HFD rats to locally inhibit neurotransmission around the upper small intestine, and found that tetracaine negated the ability of rapamycin to increase glucose infusion rate and lower glucose production during the pancreatic clamps, independent of changes in glucose uptake (Fig. 4g, h and Supplementary Fig. 4c, d). Tetracaine alone had no effects (Fig. 4g, h and Supplementary Fig. 4c, d). Next, we repeated the studies in HFD rats that received either hepatic vagal branch vagotomy (HVAG) or sham operation. HVAG vs. sham rats failed to response to upper small intestinal rapamycin infusion that led to an increase in glucose infusion rate and reduction of glucose production (Fig. 4g, h and Supplementary Fig. 4c, d). Taken together, our findings indicate that neuronal innervation of the upper small intestine and the hepatic vagal innervation are necessary for the preabsorptive effect of upper small intestinal rapamycin infusion to lower glucose production, thereby revealing a gut–brain axis that is necessary for upper small intestinal mTOR inhibition to lower hepatic glucose production in HFD rats.

### Small intestinal mTOR inhibition lowers glucose in diabetes

**Fig. 5:**
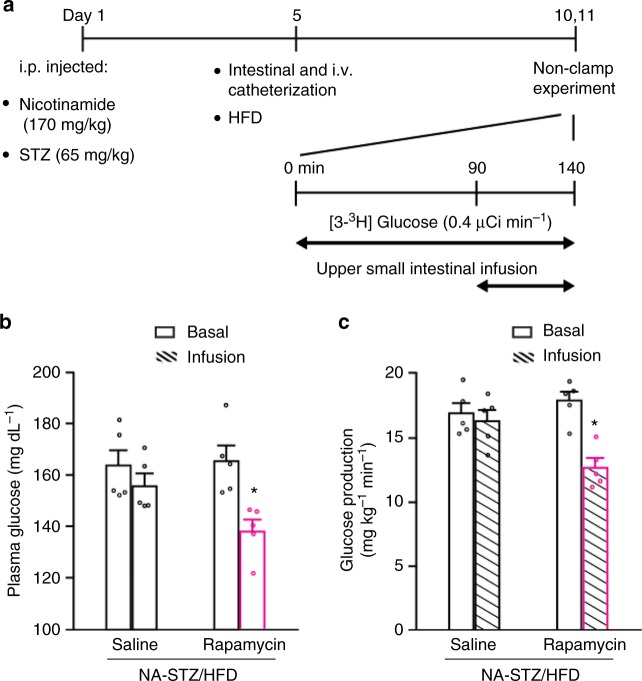
Inhibition of upper small intestinal mTOR lowers glucose in diabetic rats. **a** Experimental procedure and non-clamp experimental protocol. **b**, **c** Plasma glucose levels (**b**) and glucose production (**c**) in NA–STZ/HFD-induced hyperglycemic rats infused with upper small intestinal saline or rapamycin. *p *<* 0.05 vs. saline as compared by unpaired *t*-test; *n* *=* 5 for each group. Values are shown as mean ± s.e.m.

To assess the glucose-lowering effect of small intestinal mTOR in diabetes, we finally infused rapamycin into the upper small intestine of a rat model with diabetes that potentially reflect the clinical conditions of people with type 2 diabetes^36^. The rats are injected with low-dose streptozotocin (STZ) and nicotinamide (NA) to prevent beta cells to generate hyperinsulinemia and compensate for HFD (maintained for 5–6 days)-induced insulin resistance (Fig. 5a). As such, these rats, consistent with previous reports^19, 20, 37^, exhibited mild hyperglycemia (Supplementary Figure 5a), elevated rates of glucose production (Supplementary Figure 5b), and with no compensatory changes in plasma insulin levels measured at basal levels [0.8 ± 0.1 ng ml^−1^ for NA-STZ injected HFD rats (*n* = 8) vs. 0.9 ± 0.1 ng ml^−1^ for saline injected chow-fed rats (*n* = 6)]. We found upper small intestinal infusion of rapamycin vs. saline for 50 min that inhibited upper small intestinal, but not hepatic, mTOR signaling (Fig. 1e–g & Supplementary Figure 1g,h) also lowered plasma glucose levels (Fig. 5b) by inhibiting glucose production (Fig. 5c) in NA-STZ injected HFD diabetic rats during unclamped conditions, suggesting that inhibiting upper small intestinal mTOR lowers glucose levels as well in diabetic conditions. We next investigate whether 6 days small intestinal-targeted mTOR inhibition lowers glucose levels by infusing rapamycin for 50 min daily into the upper small intestine for 6 days. An independent group of rats received NA/STZ injection with a starting blood glucose levels (127 ± 4 mg/dl, *n* = 14) on day 1 (Fig. 5a), HFD and catheterization on day 5 (Fig. 5a), and were hyperglycemic (173 ± 5 mg/dl, *n* = 14) on day 10 (Fig. 5a). After 6 days of daily-repeated 50 min upper small intestinal infusion that started on day 10 while HFD were maintained, rapamycin vs. saline-infused rats had lowered blood glucose levels (Supplementary Fig. 5c), in parallel to an inhibition of mTOR signaling in the upper small intestine but also the liver (Supplementary Fig. 5d & e). Thus, although 6 days daily 50-min administration of upper small intestinal rapamycin (1 µg/min per day; ~ 165 µg/kg) inhibited upper small intestinal mTOR and lowered glucose levels in diabetic rats, these effects occurred in the presence of hepatic mTOR inhibition that is likely due to the absorption of rapamycin into the circulation and liver over 6 days. It is important to note, however, that glucose production during non-insulin-stimulated conditions is not altered in the HFD-hepatic-specific S6K knockdown vs. HFD-control rodents^27^ and that the NA-STZ injected HFD diabetic rats used herein exhibit basal insulin levels (see above). Interestingly, a daily intraperitoneal injection of rapamycin at 1000 µg/kg (~ 6× higher dosage than the current study) for 6 days instead induces insulin resistance and impairs glucose tolerance in mice^38^. This difference in glucoregulatory control as compared to the current study is likely (although clearly remains to be investigated) due to the difference in rapamycin dosage as high doses of rapamycin inhibits mTORC2^39^, and mTORC2 inhibition is documented to induce insulin resistance^40^.

Nonetheless, our data collectively illustrate that direct inhibition of mTOR in the upper small intestine is sufficient to lower glucose production and plasma glucose levels in rodents with HFD-induced insulin resistance as well as hyperglycemia, and is necessary for the glucose-lowering effect of metformin.

## Discussion

We here discovered, via chemical (i.e., rapamycin) and molecular-loss/gain-of function approaches, that upper small intestinal-targeted mTOR inhibition is necessary and sufficient to lower glucose production and restore glucose homeostasis in HFD-induced insulin resistance or type 2 diabetic rodents in vivo. In addition to the pathological effect of mTOR activation in causing hepatic and muscle insulin resistance in rodents and humans^10, 11, 41, 42^ and the fact that acute oral delivery of rapamycin inhibits mTOR and improves insulin sensitivity in the muscle of humans^14^, these results strengthen the notion in targeting the mTOR-dependent pathway in the upper small intestine to achieve metabolic benefits in metabolic syndromes.

The glucose-lowering effect of upper small intestinal-targeted acute effect of rapamycin was confirmed to be preabsorptive. Although acute (~ 60 min) rapamycin application in parallel reverses insulin resistance^26, 43–45^, chronic rapamycin supplication instead induces insulin resistance^40, 46, 47^. Such a chronic outcome of rapamycin has been referred to as pseudo-diabetic conditions^5, 6, 8^. The underlying mechanisms responsible for the divergent effect of rapamycin remain elusive and the duration of mTOR inhibition incurred by rapamycin maybe of critical importance. The acute effect of rapamycin relies largely on the formation of mTORC1, while the chronic effect of rapamycin is mediated by both mTORC1 & 2^25, 40, 45, 48, 49^. The currently described glucose-lowering effect of (A) rapamycin is achieved when rapamycin was delivered to the upper smal intestine for only 50 min, and (B) viral-mediated inhibition of upper small intestinal mTOR is seen after a short-term 3 days transduction protocol, and that rapamycin and dn/ca-mTOR did not alter pAKT Ser473 (a target of mTORC2^50^) in the current experimental conditions. Together with the fact that rapmacyin and/or Ad-dn-mTOR inhibits upper small intestinal phosorylation of S6K, S6, and 4EBP1 (downstream signaling effectors of mTORC1) (Fig. 1e–g; Fig. 2g, h) in parallel to a reduction in glucose production (Fig. 1d, Fig. 2e) and that inhibiting mTORC2 induces insulin resistance^40^, we put forward a working hypothesis that mTORC1 inhibition in the upper small intestine is sufficient to lower glucose levels. Dissecting the relative contribution of upper small intestinal mTORC1 & 2 in both acute and chronic control of glucose (and lipids and energy) homeostasis via genetic, molecular, and/or pharmacological approaches, as well as testing the therapeutic and translational relevance of long-term selective upper small intestinal mTOR inhibition in chronic HFD-induced obese and diabetic rodents, remain as research priorities. In addition, the relative contribution of glucose production by the liver, kidneys, and/or intestine to the whole-body glucoregulatory control of upper small intestinal mTOR inhibition remains to be assessed.

Upper small intestinal mTOR inhibition is also found to be necessary for the glucose-lowering effect of metformin in HFD rodents, but is not required for (or does not lie downstream of) direct short-term activation of upper small intestinal AMPK to lower glucose production. Although previous studies have documnted that direct AMPK activation inhibits mTOR both in vitro^31^ and in vivo^32^, and that exercise-induced activation of muscle AMPK reverses diet-induced mTOR over-stimulation and muscle insulin resistance in rodents^51^, the difference in observations vs. the current study may result from the duration of AMPK activation (chronic vs. acute) and experimental settings such as muscle vs. intestine. However, the fact that upper small intestinal metformin-AMPK axis does not inhibit mTOR to regulate glucose homeostasis is consistent with the view that metformin inhibits mTOR independent of AMPK signaling^22, 24^. Our unexpected discovery of the non-overlap impact of AMPK and mTOR signaling in the upper small intestine raises the possibility that targeting the mTOR-dependent pathway in the small intestine represents a unique strategy to lower glucose levels in diabetes.

Although at the level of the upper small intestine acute mTOR inhibition may not be required for AMPK activation to lower glucose production, a gut–brain axis is still necessary for the acute glucoregulatory effect of both AMPK activation and mTOR inhibition in HFD rodents. In parallel, although the mechanistic signaling pathways of the gut–brain neuronal relay remains to be defined, the GLP-1 receptor signaling at the small intestine, or specifically the vagal afferent that innervate the intestine, has been implicated to be necessary for nutrient sensing^52^ or D-allulose^53^ to regulate whole-body metabolic homeostasis. We put forward a working hypothesis, but clearly warrants future investigation, that upper small intestinal mTOR inhibition activates a gut GLP-1 receptor-dependent neuronal network to regulate glucose homeostasis.

In summary, our present findings indicate that short-term upper small intestinal mTOR inhibition exerts beneficial effects on glucose metabolism in HFD and diabetic rodents. More importantly, this study improves our understanding of small intestinal metformin action, illustrates an unique intestinal mTOR-dependent pathway in glucose homeostasis, and lays a foundation for the development of gut-targeted glucose-lowering therapies.

## Methods

### Animals

Eight-week-old male Sprague-Dawley rats (250–270 g) were obtained from Charles River Laboratories (Montreal, QC, Canada) and maintained on a 12-h light-dark cycle, and had ad libitum access to drinking water and regular chow or HFD. The regular chow (Teklad Diet 7002, Harlan Laboratories, Madison, WI) contained 18% fat, 33% protein, and 49% carbohydrate content (3.1 kcal g^−1^ total metabolizable energy), whereas the 10% lard-oil-enriched HFD (TestDiet 571 R, Purina Mills, IN, USA) contained 34% fat, 22% protein, and 44% carbohydrate (3.9 kcal g^−1^ total metabolizable energy). Rats were randomly assigned into various diet and treatment groups as described below and no rats were excluded unless otherwise indicated. All animal protocols were reviewed and approved by the Institutional Animal Care and Use Committee at the UHN in accordance with the Canadian Council on Animal Care guidelines.

### Surgery

All surgeries were performed 4 days prior to the clamp experiments, unless stated otherwise. An upper small intestinal catheter was placed ~ 6 cm distal to the pyloric sphincter for infusion purposes, while carotid artery and jugular vein cannulations were performed for sampling and infusion during the clamp. A separate group of rats received hepatic vagotomy procedures immediately prior to upper small intestinal cannulations^16^. Rats were individually housed and received regular chow diet during the recovery period unless indicated otherwise. Body weight and food intake were monitored daily and rats that did not recover back to ~90% of the pre-surgical weights were excluded from the study. All in vivo infusion studies described below were performed in conscious and unrestrained rats.

### Treatments

The following treatments were infused into the lumen of the upper small intestine during the in vivo experiments at a rate of 0.01 ml min^−1^ for 50 min: (1) saline; (2) rapamycin (50 μg; 1 μg min^−1^; ~165 μg kg^−1^; Millipore, Temecula, CA, USA); (3) metformin (200 mg kg^−1^, Sigma-Aldrich, St. Louis, MO, USA); (4) tetracaine (0.01 mg min^−1^, Sigma-Aldrich); and/or (5) A769662 (3 mg kg^−1^, Tocris Bioscience, Bristol, UK). The amount of rapamycin was chosen based on a previous study whereby direct ICV administration of 50 μg of rapamycin alter food intake in rodents^54^. In addition, given that our rats on average weight 0.3 kg at the time of the experiments, each rat then received an approximate 165 μg kg^−1^ of rapamycin and such dose is comparable to what is used in an human study whereby a single oral rapamycin treatment (80 μg kg^−1^) increases insulin sensitivity in humans^14^. The metformin (200 mg kg^−1^), tetracaine (0.01 mg min^−1^), and A769662 (3 mg kg^−1^) doses were based on our previous upper small intestinal-targeted infusion studies^19^.

### Virus injection

A subset of rats also received an upper small intestinal adenovirus injection prior to the insertion of the intestinal cannula, as established previously^30^. Briefly, 6 cm of the upper small intestine (6–12 cm distal to the pyloric sphincter) was isolated and ligated at both ends, flushed with saline, and 20 μl of adenovirus encoding the dominant-negative-acting mTOR-mutated protein (Ad-dn-mTOR; 2.4 × 10^8^ PFU ml^−1^), constitutively acting mTOR-mutated protein (Ad-ca-mTOR; 1.1 × 10^8^ PFU ml^−1^), and luciferase protein (Ad-Luc, 1.8 × 10^8^ PFU ml^−1^) dissolved in 180 μl of saline (total 200 μl) was administered via a 23-gauge needle directly into the upper small intestinal lumen. After 20 min, ligation sutures were removed, and a catheter was inserted in the site of the virus injection as described above. All mTOR constructs have an NH_2_-terminal AU1 epitope tag and the dn-mTOR variant contains an Asp^2338^ to Ala mutation that renders it catalytically inactive^28^. The ca-mTOR variant is a mutant form of mTOR that was generated by deleting amino acids 2430–2450 with ~ 5-fold higher kinase activity than that of wild type mTOR when assayed in vitro^28, 29^.

### Three-day HFD-fed model

Adenovirus injected rats were placed on a HFD on the same day of intestinal and vascular cannulations, and all other rats were placed on HFD one day following intestinal and vascular cannulations. Rats were maintained on HFD for 3 days, which results in hepatic insulin resistance^55^ and upper small intestinal lipid-sensing defects^16^. Food intake was monitored daily and rats that were hyperphagic and consumed more calories than rats receiving regular chow were included in the study.

### NA–STZ–HFD-induced diabetic model

Rats were treated with a single injection of nicotinamide (170 mg kg^−1^ i.p.) followed 15 min later by a single injection of streptozotocin (65 mg kg^−1^ i.p.). Four days later, upper small intestinal and vascular cannulations were performed, and rats were placed on a lard-oil-enriched HFD to induce insulin resistance. Rats that were extremely hyperglycemic (blood glucose level > 300 mg dl^−1^) were excluded from the study. Five to six days after surgery and HFD, rats were subjected to the basal [3–^3^H] glucose infusion protocol and upper small intestinal rapamycin infusion after a 4–6 h fast. Another set of diabetic rats had an additional 5 days of HFD while receiving daily upper small intestinal rapamycin to a total of 6 days daily rapamycin infusion.

### Pancreatic (basal insulin) euglycemic clamp procedures

Rats were fasted for ~ 4–6 h before the clamp experiment. The clamp procedure (*t* = 200 min total) was performed in unrestrained rats in vivo. At the onset of the experiment (*t* = 0 min), a primed intravenous infusion of [3-^3^H] glucose (Perkin Elmer; 40 µCi bolus; 0.4 µCi min^−1^) was started and continued throughout (*t* = 200 min) to allow for measurement of glucose kinetics using the tracer-dilution methodology. The clamp was started at *t* = 90 min, where somatostatin (3 µg kg^−1^ min^−1^) was infused intravenously to inhibit endogenous insulin and glucagon secretion, and simultaneously, insulin was infused at a dose of 1.2 mU kg^−1^ min^−1^ for the pancreatic (basal insulin) clamp, along with a variable 25% glucose infusion that was periodically adjusted to maintain euglycemia (from *t* = 120 to *t* = 200 min). Plasma samples were obtained every 10 min to determine the specific activity of [3-^3^H] glucose and measure insulin levels. At the end of the experiments, rats were anesthetized and tissue samples were collected, immediately flash frozen, and stored at –80 °C until use.

### Basal [3-^3^H] glucose infusion protocol (non-clamp conditions)

Rats were fasted for ~ 4–6 h before the experiment. These studies were performed in the diabetic model described above. The infusion experiment was 140 min in duration, and performed in unrestrained rats in vivo. A primed continuous infusion of [3-^3^H] glucose was started at *t* = 0 min and continued until *t* = 140 min. Upper small intestinal infusions of treatments outlined below were started at *t* = 90 min and continued throughout the experiment. Plasma samples were collected every 10 min from *t* = 60 min to *t* = 90 min and from *t* = 100 min and *t* = 140 min to determine plasma glucose levels and [3-^3^H] glucose specific activity.

### Tissue collection and preparation for western blotting

Immediately following termination of the experiments, rats were anaesthetized, 6 cm of the upper small intestine (6–12 cm distal to the pyloric sphincter) was removed and flushed with PBS containing protease inhibitors, the mucosa was immediately scraped and separated from the smooth muscle, and the liver was freeze clamped using steel tongs pre-cooled in liquid nitrogen. The tissues were lysed on ice with a handheld homogenizer in a lysis buffer containing 50 mM Tris-HCl (pH 7.5), 1 mM EGTA, 1 mM EDTA, 1% (w/v) Nonidet P40, 1 mM sodium orthovanadate, 50 mM sodium fluoride, 5 mM sodium pyrophosphate, 0.27 M sucrose, 1 μM dithiothreitol (DTT) and 2 × protease inhibitor cocktail (Roche). The protein concentration of homogenized tissues was determined using the Pierce 660 nm protein assay (Thermo Scientific).

### Cell culture

Adenoviruses expressing ca-mTOR and dn-mTOR were generated, amplified, and purified as previously been shown^28^. For in vitro experiments, HEK293 cells (originally sourced from ATCC (CRL-1573)) and PC12 cells (originally sourced from ATCC (CRL-1721)) were seeded (1 × 10^6^ cell/well) onto 6-well plates and 4 h later were infected with Ad-ca-mTOR (0.08 MOI) or Ad-dn-mTOR (200 MOI), respectively, and Ad-Luc with the same concentration of mTOR viruses in that cells. After 24 h viral transduction, the cell media was changed and HEK293 cells were treated with 10 mM metformin in complete media for at least 12–16 h while PC12 cells were incubated with serum free media for 16 h and then treated with serum for 1 h or 2.6 nM IGF1 for 15 min or 0.4 mM leucine for 10 min. We used 10 mM metformin based off our previous study^19^ with HEK293 cells, where this dose activates AMPK. Cells were lysed in a buffer containing 50 mM Tris-HCl (pH 7.5), 1 mM EGTA, 1 mM EDTA, 1% (w/v) Nonidet P40, 1 mM sodium orthovanadate, 50 mM sodium fluoride, 5 mM sodium pyrophosphate, 0.27 M sucrose, 1 μM Dithiotritolo (DTT) and 2 × protease inhibitor cocktail (Roche). The Pierce 660 nm protein assay (Thermo Scientific) was used for measuring of protein concentration. All cell lines were routinely tested for mycoplasma contamination. The treatment duration and dose of IGF1 and leucine were chosen based on previous studies whereby 15 min 2.6 nM IGF1 in IGF-1R/IRS1 cells^56^ and 10 min 0.4 mM leucine in HeLa cells^57^ significantly increases the phosphorylation of S6K protein.

### Western blotting

Five to fifteen microgram of cell culture or fifty microgram of tissues lysates (prepared as described above) were subjected to electrophoresis on 10% acrylamide gels and transferred to nitrocellulose membranes. The membranes were incubated for 1 h with blocking buffer (either TBS-T containing 5% (w/v) BSA or 5% skim milk). The membranes were then incubated with the indicated primary antibodies: anti-β-actin (1:32,000, Sigma-Aldrich, Cat #A1978), anti-p70 S6K (1:1000, Cell Signaling, MA, USA #9202), anti-p-p70 S6K Thr389 (1:1000, Cell Signaling, #9234), anti-S6 ribosomal protein 5G10 (1:1000, Cell Signaling, #2217), anti-p-S6 ribosomal protein Ser 235/236 (1:1000, Cell Signaling, #4857), anti-p-4EBP1 Ser65 (1:1000, Cell Signaling, #9451), anti-AKT (1:1000, Cell Signaling, #9272), anti-p-AKT Ser 473 (1:1000, Cell Signaling, #9271), anti-p-AKT Thr308 (1:1000, Cell Signaling, #4056), anti-ACC (1:1000, Cell Signaling, #3676), anti-p-ACC Ser79 (1:1000, Cell Signaling, #3661), anti-raptor (1:1000, Cell Signaling, #2280), anti-p-raptor Ser792 (1:1000, Cell Signaling, #2083), anti-AMPKα (1:1000, Santa Cruz Biotechnology, #sc25792), anti-p-AMPKα Thr172 (1:1000, Cell Signaling, #2535 s), anti-mTOR (1:1000, Cell Signaling, #2972), and anti-AU1 (1:1000, Biolegend Inc, CA, USA, #901901), diluted in the blocking buffer for 16 h at 4 °C. The membranes were washed three times with TBS-T and incubated with the appropriate secondary HRP-conjugated antibodies (diluted 1:4,000 for all but 1:32,000 for β-actin in 5% skim milk) at room temperature for 1 h. Finally, the membranes were washed in TBS-T five times for 5 min each, and the signal was detected using enhanced chemiluminescence reagent (Pierce, IL, USA). Immunoblots were developed with MicroChemi 4.2 chemiluminescent imaging system (DNR Bio-Imaging Systems, Jerusalem, Israel), and protein levels were quantified by GelQuant image analysis software (version 13.1, DNR Bio-Imaging Systems). See Supplementary Fig. 6–10 for uncropped blots.

### Biochemical analysis

Plasma glucose concentrations were measured by the glucose oxidase method (Glucose Analyzer GM9, Analox Instruments, Lunenburg, MA), and blood glucose concentrations were measured using a glucometer (OneTouch Ultra2, LifeScan Europe, Zug, Switzerland) with blood taken by tail-prick. Plasma insulin levels were determined by radioimmunoassay (Millipore Canada Ltd, Etobicoke, ON).

### Quantitative PCR (qPCR) analysis

Approximately 75 mg of the mucosal layer of the upper small intestine (~ 6–12 cm distal from the pyloric sphincter) was separated from the smooth muscle layer immediately following dissection. Mucosal scrapings were homogenized in lysis buffer (Ambion) using a PowerGen-125 homogenizer (Thermo Fisher Scientific, Toronto, ON) and centrifuged at 12,500 × *g* for 5 min, and RNA was isolated using the Ambion PureLink RNA Mini Kit per kit guidelines (Thermo Fisher Scientific). RNA was quantified by measuring the absorbance at 260 and 280 nm using Cytation 5 imaging reader (BioTek, Winooski, VT). Five μg of RNA was subjected to DNase digestion (Roche, Mannheim, Germany) at room temperature for 10 min and terminated by the addition of 25 mM EDTA and incubating at 70 °C for 15 min. cDNA was generated using the SuperScript Vilo cDNA Synthesis Kit as per the manufacturer’s instructions (Invitrogen, Carlsbad, CA, USA). qPCR was performed using 500 ng of cDNA, TaqMan Gene Expression master mix, and TaqMan primers (Thermo Fisher Scientific) for rat ribosomal protein 18s (*Rps 18;* Assay ID: Rn01428913_gH), rat *G6Pase* (*G6pc;* Assay ID: Rn00689876_m1) or rat *PEPCK1* (*Pck1;* Assay ID: Rn01529014_m1) using a QuantStudio 7 Flex qPCR machine (Applied Biosystems). Relative gene expression was calculated using the ΔΔCt method in QuantStudio Real-Time PCR software (version 1.2, Applied Biosystems, Thermo Fisher Scientific), where each sample was normalized to 18s as the reference gene.

### Statistical analysis

The sample size for each group was chosen based on study feasibility and prior knowledge of statistical power from previously published experiments. All statistical analysis was performed using GraphPad Prism (version 6.01, GraphPad, La Jolla, CA, USA). All measurements were taken from distinct samples. Unpaired Student’s *t*-tests (two-sided with 95% confidence level) were performed in the statistical analysis of two groups. Where comparisons were made across more than two groups, one-way analysis of variance (ANOVA) with Tukey post hoc test was performed. Differences were considered significant at *p* < 0.05. For the clamp experiments, the time period of 60–90 min was averaged for the basal condition, and the time period of 180–200 min was averaged for the clamp condition. For non-clamp experiments, the time period 60–90 min was averaged for the basal condition, and the time period from 130 to 140 min was averaged for treatment conditions. All numerical results are presented as mean ± s.e.m.

### Reporting summary

Further information on experimental design is available in the Nature Research Reporting Summary linked to this article.

## Supplementary information

Supplementary Information

Reporting Summary

## Data Availability

All the relevant data are available from the authors on reasonable request and/or are included within the manuscript (and its Supplementary Information files).
