# Supplementary material for: Inhibition of upper small intestinal mTOR lowers plasma glucose levels by inhibiting glucose production
Source: Nat Commun. 2019 Feb 12;10:714. doi: 10.1038/s41467-019-08582-7 (PMC6372624; doi:10.1038/s41467-019-08582-7)
Supplement: Supplementary file 1 — Supplementary Information [file 41467_2019_8582_MOESM1_ESM.pdf]

Inhibition of upper small intestinal mTOR lowers plasma glucose levels by inhibiting glucose production (Waise et al.)

Supplementary information

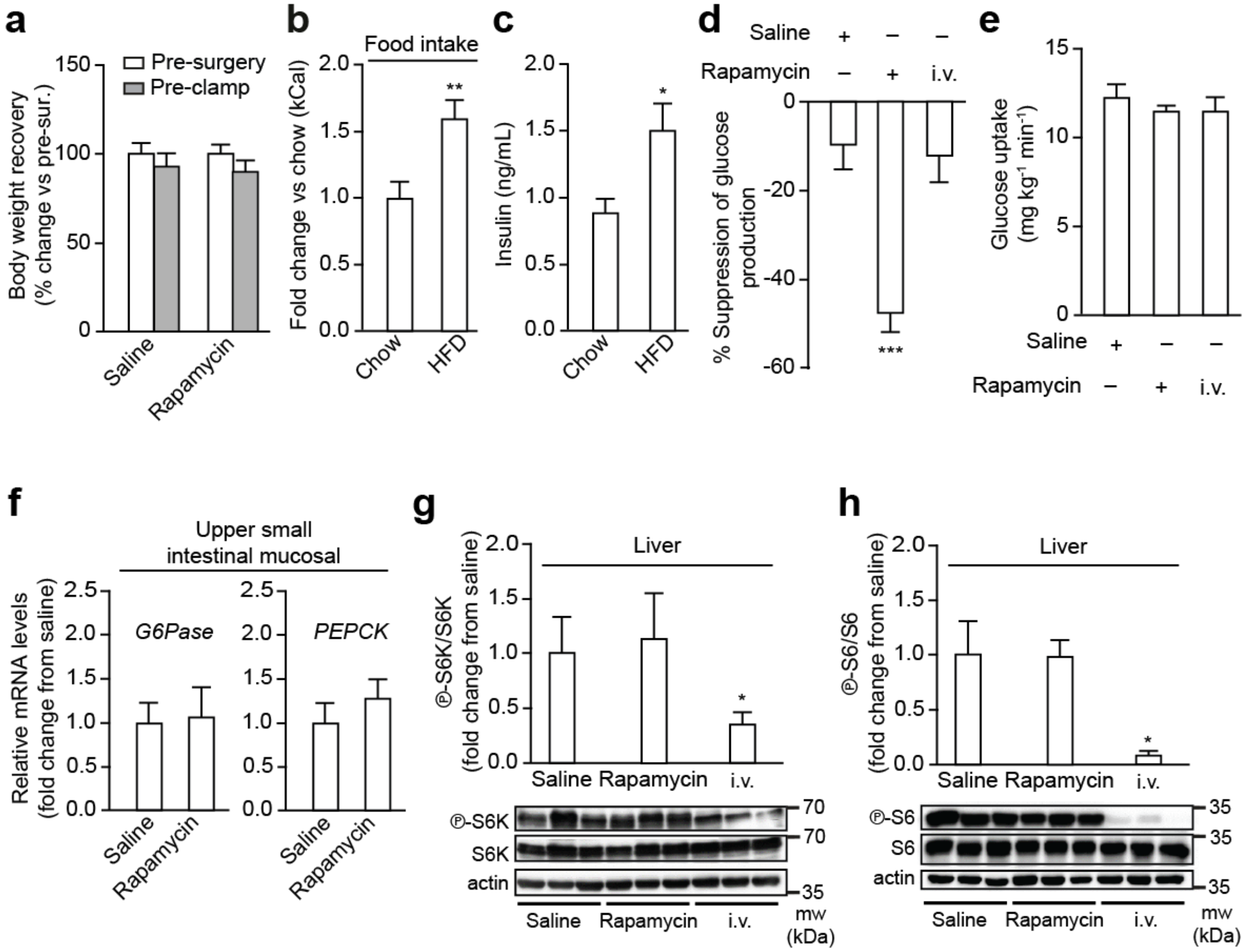

Supplementary Figure 1. Body weight, food intake, insulin level, glucose kinetics, western blots for liver tissue, and intestinal gluconeogenic gene expression analysis relating to Figure 1. a, Body weight on the day of surgery and on the day of clamp for three days HFD rats given a saline or rapamycin upper small intestinal infusion (n = 10 for each group). Data are presented as percent recovery over the pre-surgical body weights. **b**, Cumulative food intake for three days prior to the clamp for HFD-fed rats (n = 20), expressed as a fold change from RC-fed rats (n = 13). $^{**}p < 0.01$ versus chow-fed group as determined by unpaired t-test. **c**, Plasma insulin level of three days normal chow-fed (n = 6), and HFD-fed (n = 6) rats. $^{*}p < 0.05$ versus chow-fed group as determined by unpaired t-test. (**d, e**) Glucose production percent suppression from basal (d) and glucose uptake (e) during the pancreatic (basal insulin) euglycemic clamp in HFD-fed rats infused with upper small intestinal saline (n = 8) or rapamycin (n = 6) or with i.v. rapamycin (n = 7). $^{*}p < 0.05$ versus all other groups as determined by ANOVA with Tukey's post hoc test. **f**, Upper small intestinal mucosal mRNA expressions of glucose-6-phosphatase (*G6Pase*) and phosphoenolpyruvate carboxykinase (*PEPCK*) in HFD rats that received 50min upper intestinal saline (n = 8 for *G6Pase*, and 7 for *PEPCK*) or rapamycin (n = 7 for *G6Pase*, and 6 for *PEPCK*) infusion. (**g, h**) Quantitative analysis and representative western blot of phosphorylated S6K (f) and S6 (g) protein expression normalized to their respective total in the liver tissue of HFD rats infused with upper intestinal saline (n = 6 for S6K, and 7 for S6) or rapamycin (n = 7) or with i.v. rapamycin (n = 6 for S6K, and 7 for S6). Actin, loading control. $^{*}p < 0.05$ versus saline as calculated by unpaired t-test. Values are shown as mean ± s.e.m.

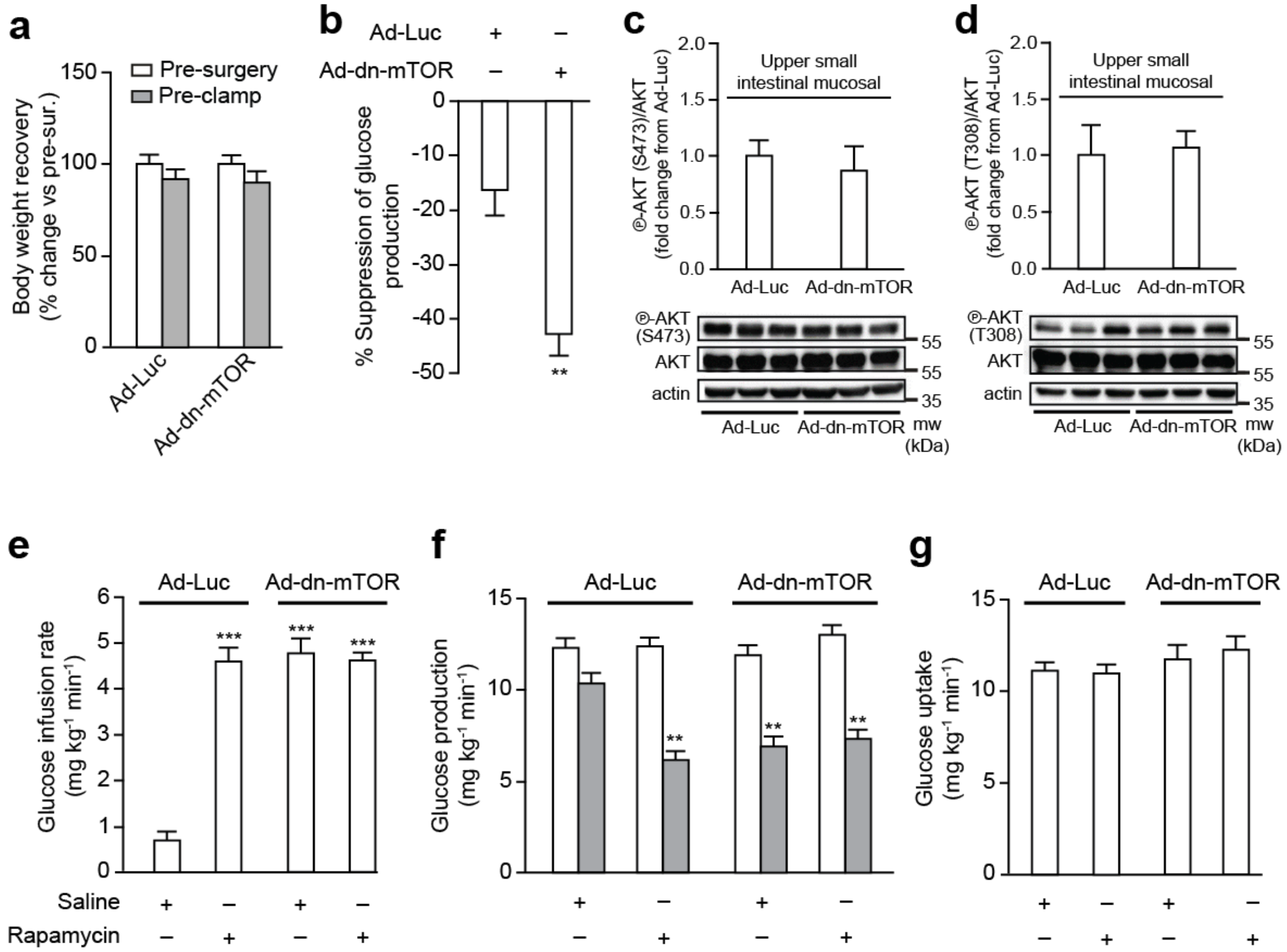

Supplementary Figure 2. Body weight, glucose kinetics, and western blots for in upper small intestinal mucosal tissues in 3d HFD rats relating to Figure 2. **a**, Body weight on the day of surgery and on the day of clamp for three days HFD rats infected with Ad-Luc or Ad-dn-mTOR (*n* = 10 for each group). Data are presented as percent recovery over the pre-surgical body weights. **b**, Glucose production percent suppression from basal during the clamp in HFD-fed rats infected with 3 d upper small intestinal Ad-Luc or Ad-dn-mTOR (*n* = 6 for each group). $^{**}p < 0.01$ versus Ad-Luc; calculated by unpaired *t*-test. **(c, d**) Quantitative analysis and representative western blot of phosphorylated AKT (S473) (c) and AKT (T308) (d) protein expression normalized to their respective total in the upper small intestinal mucosal tissue of HFD rats infected with either Ad-Luc (*n* = 8 for AKT473, and 7 for AKT308) or Ad-dn-mTOR (*n* = 6/group) for three days. Actin, loading control. **(e-g**) The glucose infusion rate (e), glucose production (f), and glucose uptake (g) during the clamp in HFD-fed rats infected with Ad-Luc or Ad-dn-mTOR and infused with either upper intestinal saline (*n* = 6) or rapamycin (*n* = 6). $^{***}p < 0.001$, $^{**}p < 0.01$ versus Ad-Luc+saline; calculated by ANOVA with Tukey’s post hoc test. Values are shown as mean ± s.e.m.

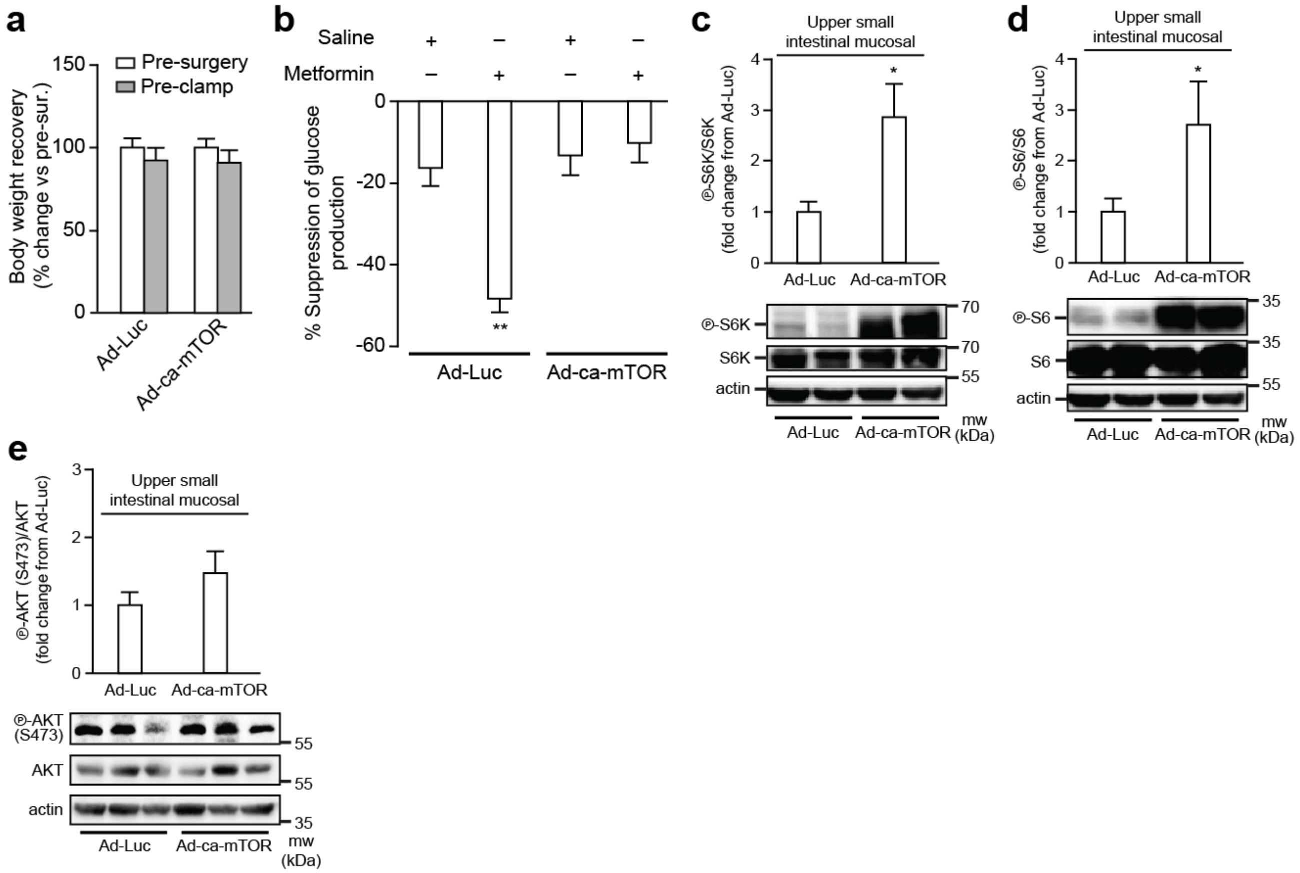

Supplementary Figure 3. Body weight, glucose kinetics, and western blots for mTOR signaling in upper small intestinal mucosal tissues in 3d HFD rats relating to Figure 3. a, Body weight on the day of surgery and on the day of clamp for three days HFD rats infected with Ad-Luc or Ad-ca-mTOR (*n* = 10 for each group). Data are presented as percent recovery over the pre-surgical body weights. **b**, Glucose production percent suppression from basal during the clamp in HFD-fed rats infected with either upper small intestinal Ad-Luc or Ad-ca-mTOR and infused with upper intestinal saline (*n* = 6 for each group) or metformin (*n* = 7 for each group). $**p < 0.01$ versus all other groups as determined by ANOVA with Tukey's post hoc test. (**c**-**e**) Quantitative analysis and representative western blot of phosphorylated S6K (c), S6 (d) and AKT (S473) (e) protein expression normalized to their respective total in the upper small intestinal mucosal tissue of HFD rats infected with either Ad-Luc (*n* = 7 for S6, and 6 for other groups) or Ad-ca-mTOR (*n* = 7 for S6, and 6 for other groups) for three days. $*p < 0.05$ versus Ad-Luc as calculated by unpaired *t*-test. Actin, loading control. Values are shown as mean ± s.e.m.

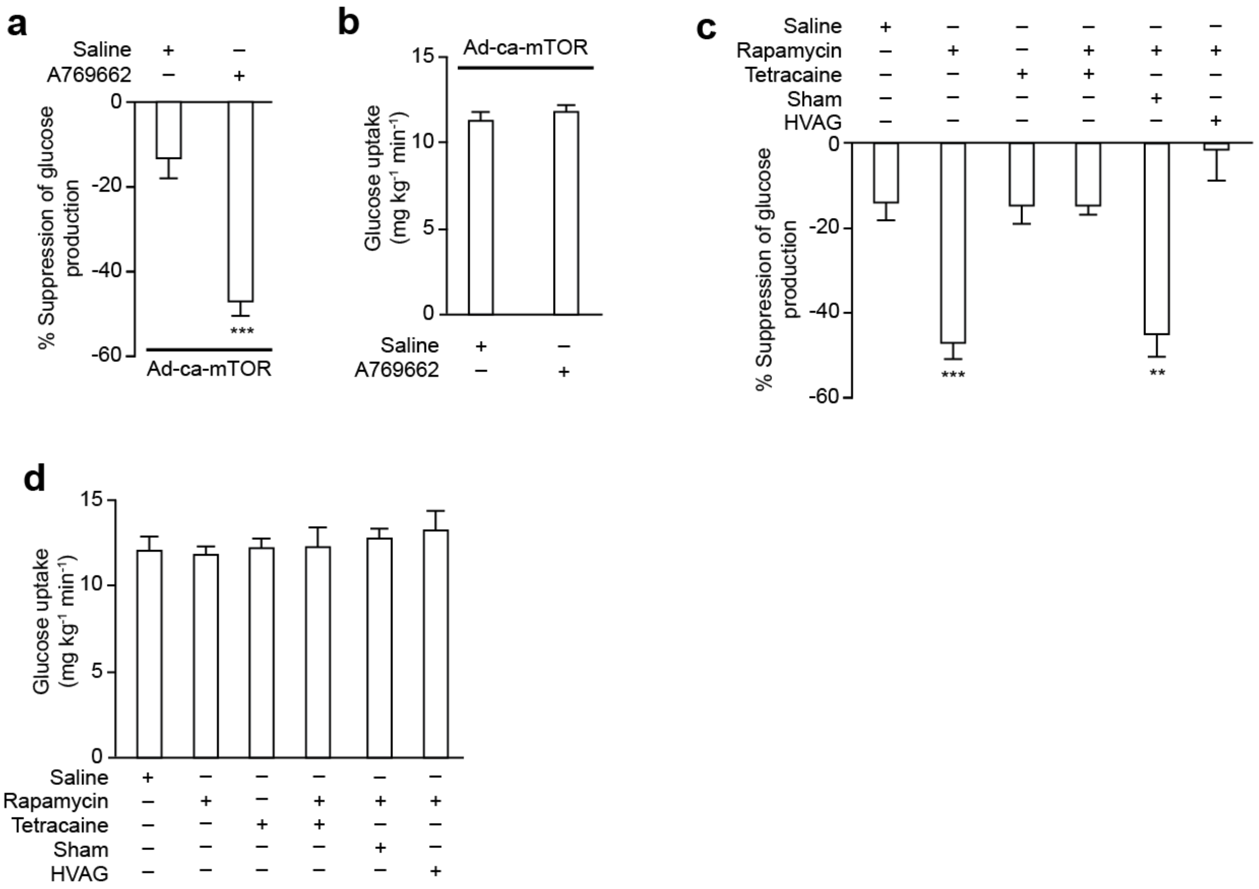

Supplementary Figure 4. Glucose kinetics in 3d HFD rats relating to Figure 4. (**a, b**) Glucose production percent suppression from basal (a), and glucose uptake (b) during the clamps in HFD-fed rats infected with upper small intestinal Ad-ca-mTOR and infused with upper intestinal A769662. ****p* < 0.001 versus saline; calculated by unpaired *t*-test (*n* = 6 for each group). (**c, d**) Glucose production percent suppression from basal (c), and glucose uptake (d) during clamps in HFD-fed rats infused with upper small intestinal saline (n = 7), rapamycin (n = 7), tetracaine (n = 5), rapamycin + tetracaine (n = 6), rapamycin after either sham (n = 5) or hepatic vagal branch vagotomy (HVAG) (n = 5). **p < 0.01, ***p < 0.001 versus saline, tetracaine, tetracaine + rapamycin, and HVAG +rapamycin as determined by ANOVA with Tukey's post hoc test. Values are shown as mean ± s.e.m.

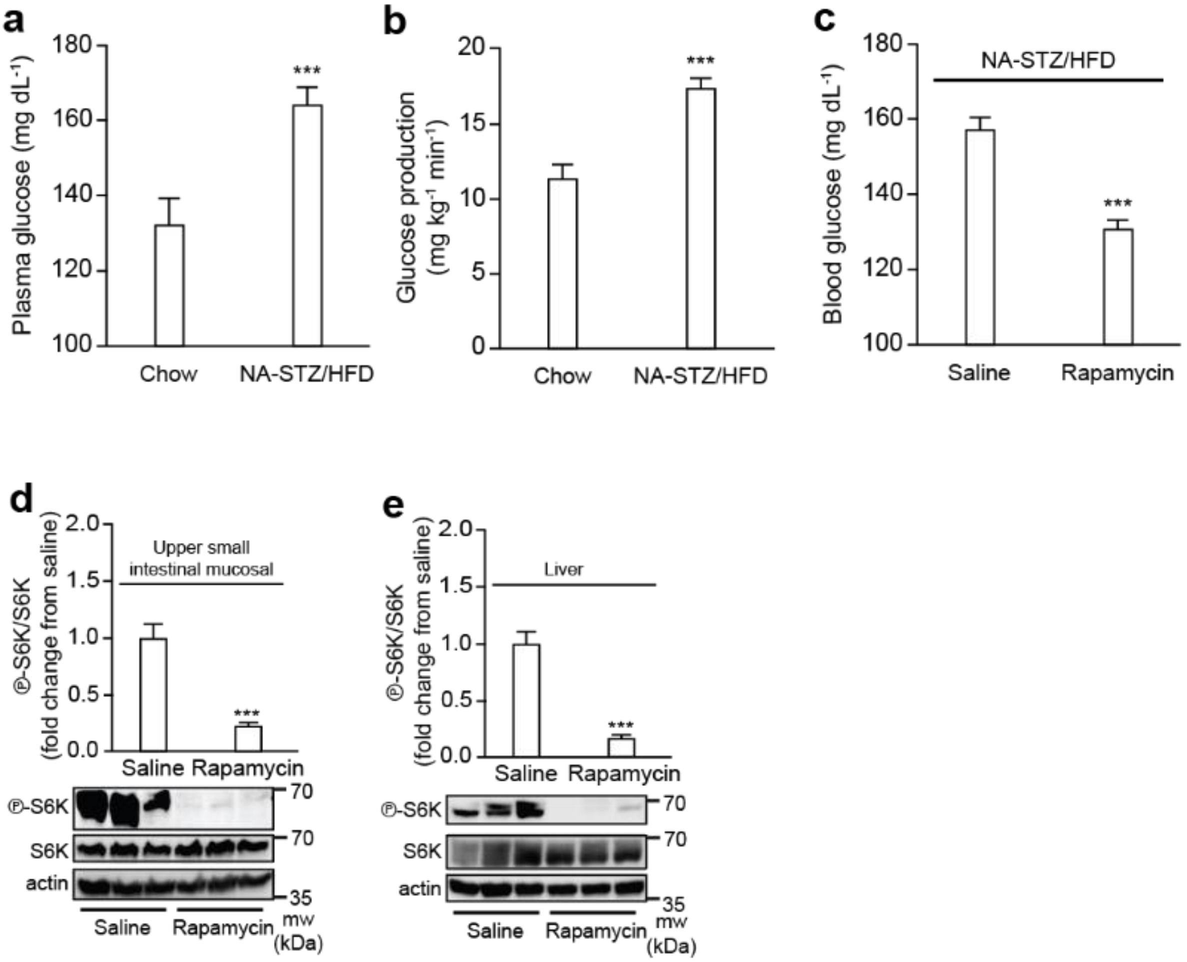

Supplementary Figure 5. Glucose levels, and western blots relating to figure 5. (**a, b**) Plasma glucose levels (**a**) and glucose production (**b**) in the basal state of normal chow-fed (n = 6), and NA-STZ/HFD hyperglycemic (n = 10) rats. ***p < 0.001 versus chow-fed group as determined by unpaired t-test. (**c**) Blood glucose levels after 6 consecutive days of 50 min upper small intestinal saline (n = 7) or rapamycin (n = 7) infusion in NA-STZ/HFD hyperglycemic rats. ***p < 0.001 versus saline group as determined by unpaired t-test. (**d, e**) Quantitative analysis and representative western blot of phosphorylated S6K protein expression normalized to their respective total in the upper small intestinal mucosal (d) and liver (e) tissue of NA-STZ/HFD hyperglycemic rats with 50 min saline (n = 6) or rapamycin (n = 6 for intestine, and 7 for liver) infusion for 6 consecutive days. *p < 0.05 versus saline as calculated by unpaired t-test. Actin, loading control. Values are shown as mean ± s.e.m.

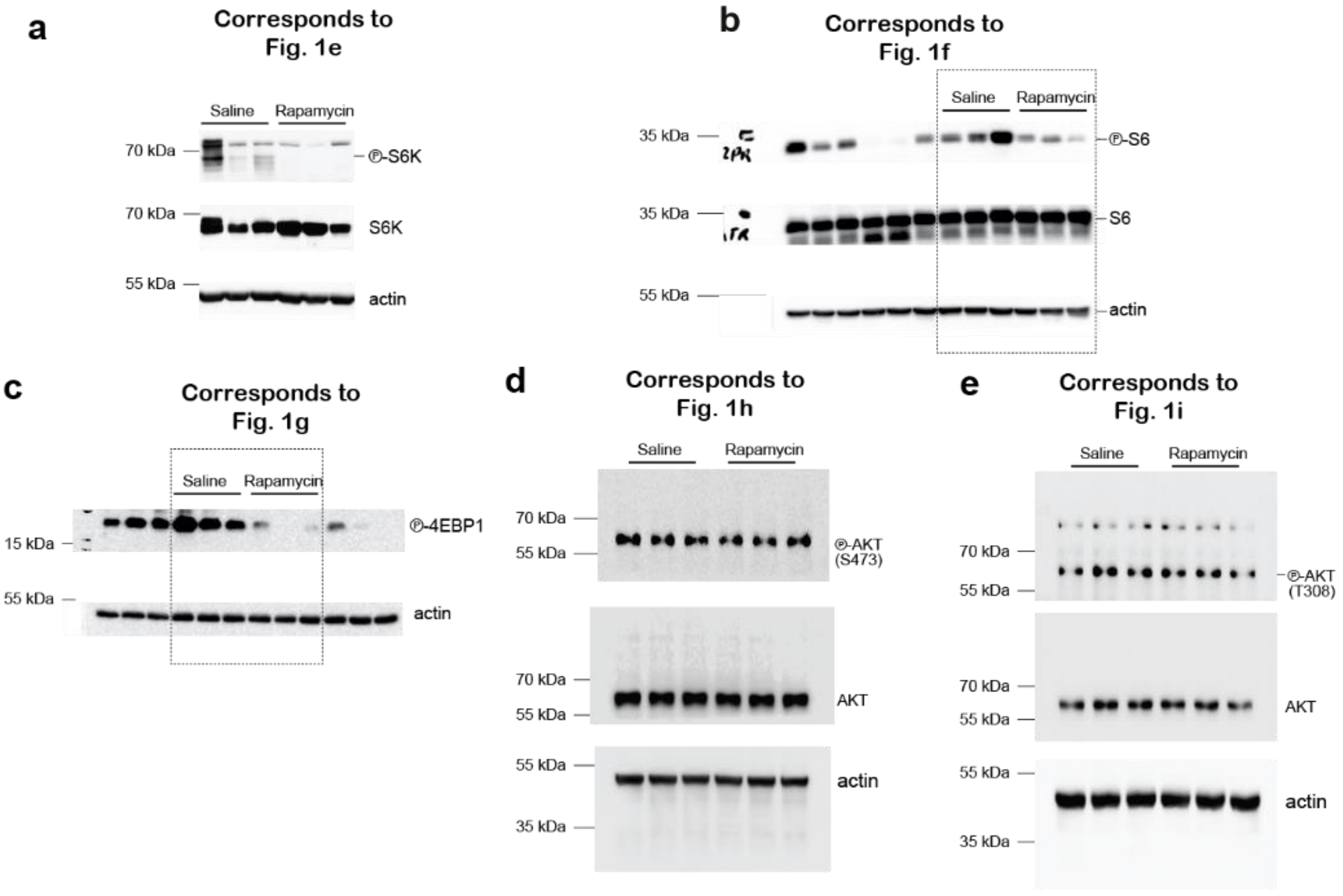

Supplementary Figure 6. Scans of uncropped Western blots. Corresponding to representative images shown in **(a)**: Fig. 1e; **(b)**: Fig. 1f; **(c)**: Fig. 1g; **(d)**: Fig. 1h; **(e)**: Fig. 1i.

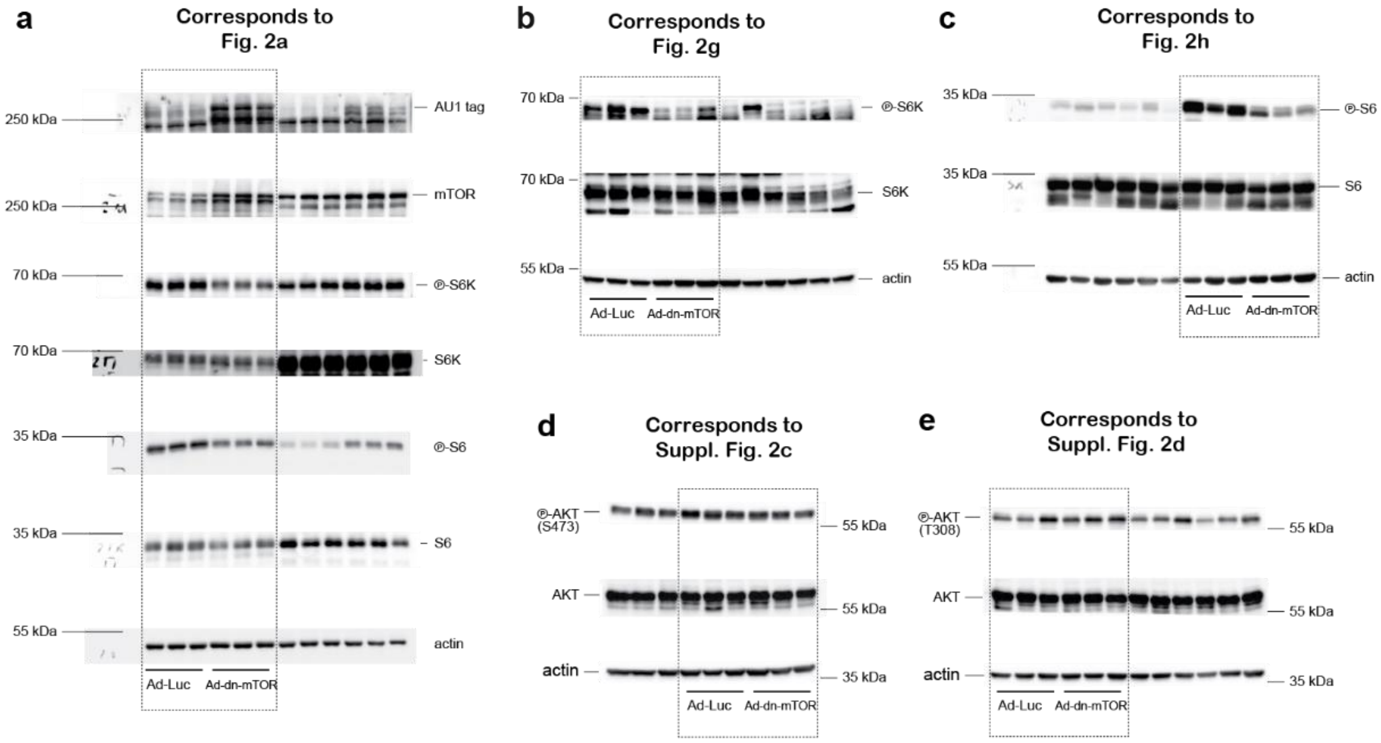

Supplementary Figure 7. Scans of uncropped Western blots. Corresponding to representative images shown in **(a)**: Fig. 2a; **(b)**: Fig. 2g; **(c)**: Fig. 2h; **(d)**: Suppl. Fig. 2c; **(e)**: Suppl. Fig. 2d.

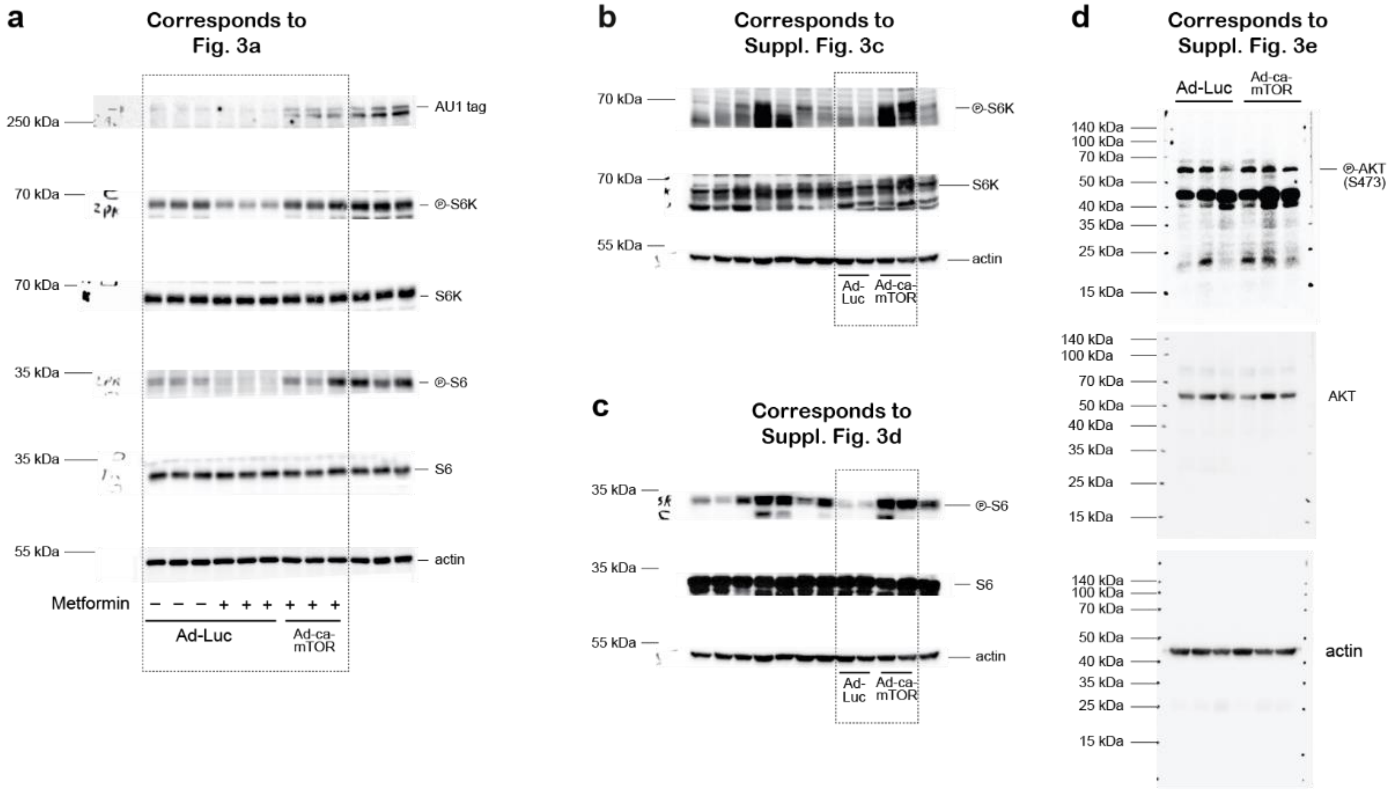

Supplementary Figure 8. Scans of uncropped Western blots. Corresponding to representative images shown in **(a)**: Fig. 3a; **(b)**: Suppl. Fig. 3c; **(c)**: Suppl. Fig. 3d; **(d)**: Suppl. Fig. 3e.

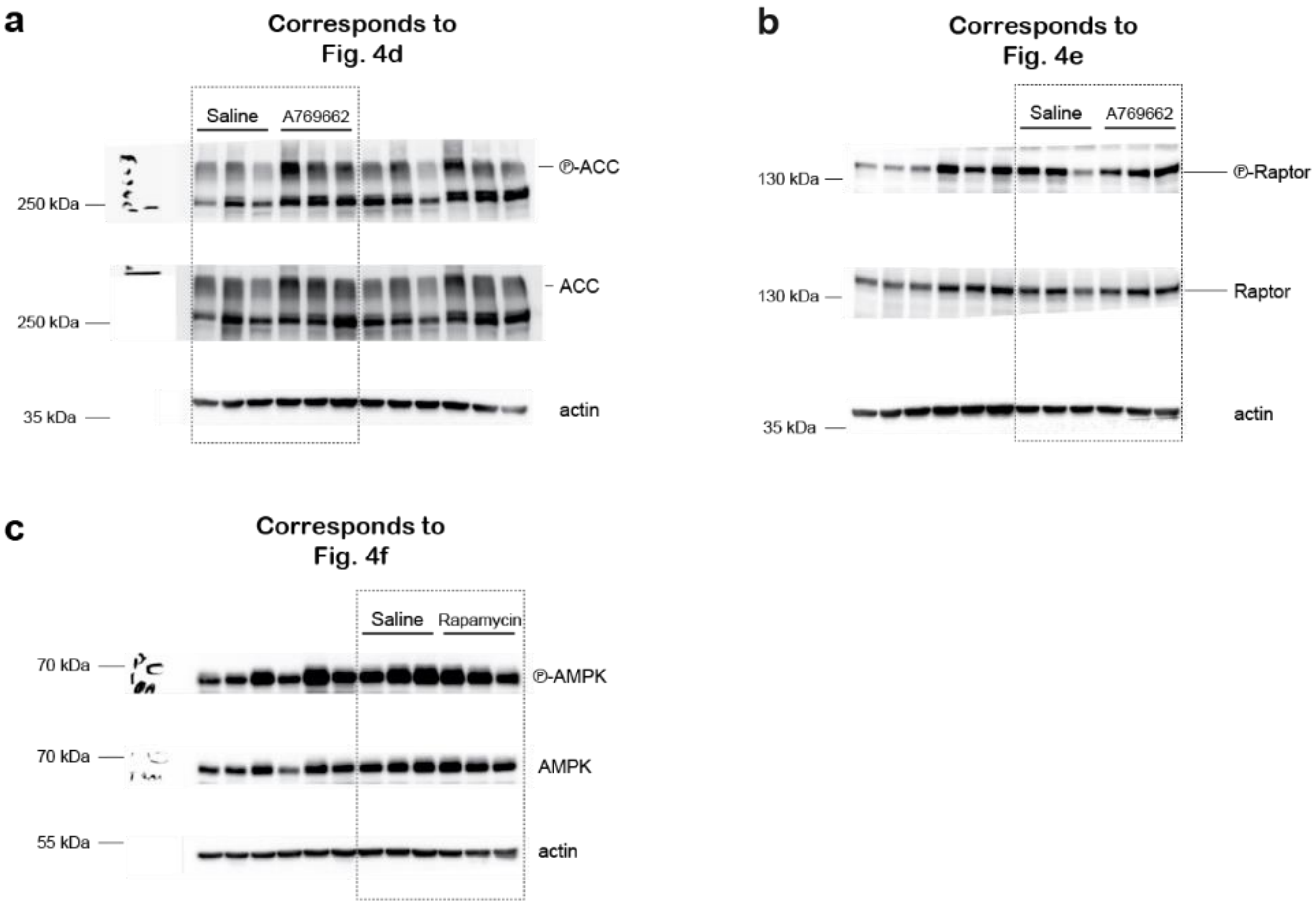

Supplementary Figure 9. Scans of uncropped Western blots. Corresponding to representative images shown in **(a)**: Fig. 4d; **(b)**: Fig. 4e; **(c)**: Fig. 4f.

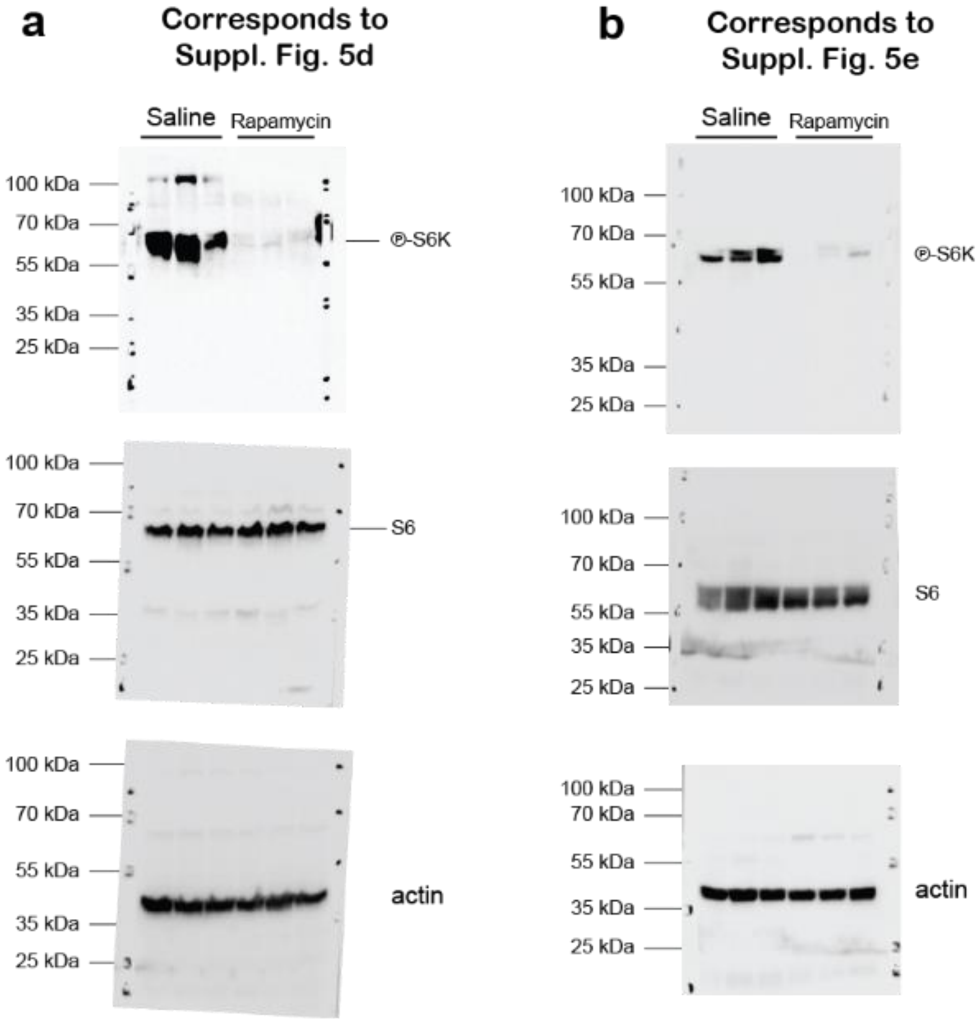

Supplementary Figure 10. Scans of uncropped Western blots. Corresponding to representative images shown in **(a)**: Suppl. Fig. 5d; **(b)**: Suppl. Fig. 5e.

Supplementary Table 1. Plasma insulin and glucose concentrations of the groups receiving upper small intestinal infusion during the clamp condition.

	HFD + saline (n = 6)	HFD + rapamycin (n = 5)
Insulin (ng/mL)	1.1 ± 0.2	1.2 ± 0.2
Glucose (mM)	7.5 ± 0.3	7.4 ± 0.7

Data are mean ± s.e.m.
