# Supplementary material for: Inhibition of upper small intestinal mTOR lowers plasma glucose levels by inhibiting glucose production
Source: Nat Commun. 2019 Feb 12;10:714. doi: 10.1038/s41467-019-08582-7 (PMC6372624; doi:10.1038/s41467-019-08582-7)
Supplement: Supplementary file 2 — Reporting Summary [file 41467_2019_8582_MOESM2_ESM.pdf]

nature research

Corresponding author(s): Dr. Tony Lam

Last updated by author(s): Jan 3, 2019

Reporting Summary

Nature Research wishes to improve the reproducibility of the work that we publish. This form provides structure for consistency and transparency in reporting. For further information on Nature Research policies, see Authors & Referees and the Editorial Policy Checklist.

Statistics

For all statistical analyses, confirm that the following items are present in the figure legend, table legend, main text, or Methods section.

n/a	Confirmed	
☐	☒	The exact sample size (*n*) for each experimental group/condition, given as a discrete number and unit of measurement
☐	☒	A statement on whether measurements were taken from distinct samples or whether the same sample was measured repeatedly
☐	☒	The statistical test(s) used AND whether they are one- or two-sided<br>*Only common tests should be described solely by name; describe more complex techniques in the Methods section.*
☐	☒	A description of all covariates tested
☐	☐	A description of any assumptions or corrections, such as tests of normality and adjustment for multiple comparisons
☒	☐	A full description of the statistical parameters including central tendency (e.g. means) or other basic estimates (e.g. regression coefficient) AND variation (e.g. standard deviation) or associated estimates of uncertainty (e.g. confidence intervals)
☐	☒	For null hypothesis testing, the test statistic (e.g. *F*, *t*, *r*) with confidence intervals, effect sizes, degrees of freedom and *P* value noted<br>*Give P values as exact values whenever suitable.*
☒	☐	For Bayesian analysis, information on the choice of priors and Markov chain Monte Carlo settings
☒	☐	For hierarchical and complex designs, identification of the appropriate level for tests and full reporting of outcomes
☒	☐	Estimates of effect sizes (e.g. Cohen's *d*, Pearson's *r*), indicating how they were calculated

Our web collection on statistics for biologists contains articles on many of the points above.

Software and code

Policy information about availability of computer code

Data collection	No software was used
Data analysis	GraphPad Prism (version 6.01, GraphPad, La Jolla, CA, USA); GelQuant image analysis software (DNR Bio-Imaging Systems, Jerusalem, Israel); MicroChemi 4.2 chemiluminescent imaging system (DNR Bio-Imaging Systems); QuantStudio Real-Time PCR software (version 1.2, Applied Biosystems, Thermo Fisher Scientific, Toronto ON Canada); Microsoft Excel 2016

For manuscripts utilizing custom algorithms or software that are central to the research but not yet described in published literature, software must be made available to editors/reviewers. We strongly encourage code deposition in a community repository (e.g. GitHub). See the Nature Research guidelines for submitting code & software for further information.

Data

Policy information about availability of data

All manuscripts must include a data availability statement. This statement should provide the following information, where applicable:

- Accession codes, unique identifiers, or web links for publicly available datasets
- A list of figures that have associated raw data
- A description of any restrictions on data availability

A full data availability statement is included in the manuscript (Page 22)

Field-specific reporting

Please select the one below that is the best fit for your research. If you are not sure, read the appropriate sections before making your selection.

☒ Life sciences ☐ Behavioural & social sciences ☐ Ecological, evolutionary & environmental sciences

For a reference copy of the document with all sections, see nature.com/documents/nr-reporting-summary-flat.pdf

Life sciences study design

All studies must disclose on these points even when the disclosure is negative.

Sample size	The exact sample sizes for each experimental group/condition are listed in figure legends. The sample size for each group was chosen on the basis of study feasibility and prior knowledge of statistical power form previously published experiments (Page 21).
Data exclusions	No data were excluded from the analyses.
Replication	All attempts at replication were successful.
Randomization	Rats were randomly assigned into various diet and treatment groups.
Blinding	The investigators involved in this study were not completely blinded during data collection and/or sample collection and analysis.

Reporting for specific materials, systems and methods

We require information from authors about some types of materials, experimental systems and methods used in many studies. Here, indicate whether each material, system or method listed is relevant to your study. If you are not sure if a list item applies to your research, read the appropriate section before selecting a response.

Materials & experimental systems

n/a	Involved in the study	
☐	☒	Antibodies
☐	☒	Eukaryotic cell lines
☒	☐	Palaeontology
☐	☒	Animals and other organisms
☒	☐	Human research participants
☒	☐	Clinical data

Methods

n/a	Involved in the study	
☒	☐	ChIP-seq
☒	☐	Flow cytometry
☒	☐	MRI-based neuroimaging

Antibodies

Antibodies used	Data provided in the manuscript
Validation	Data provided in the manuscript

Eukaryotic cell lines

Policy information about cell lines

Cell line source(s)	HEK293 cells originally sourced from ATCC (CRL-1573),<br>PC12 cells originally sourced from ATCC (CRL-1721)
Authentication	Cell lines were checked regularly for contamination but not authenticated before use.
Mycoplasma contamination	Cell lines were routinely tested for mycoplasma contamination.
Commonly misidentified lines (See ICLAC register)	No commonly misidentified cell lines were used.

Animals and other organisms

Policy information about studies involving animals; ARRIVE guidelines recommended for reporting animal research

Laboratory animals	Male Sprague-Dawley rats (250-270 g upon arrival, approximately 8 weeks of age) were obtained from Charles River Laboratories.
Wild animals	The study did not involve wild animals
Field-collected samples	The study did not involve samples collected from the field
Ethics oversight	Institutional Animal Care and Use Committee of the University Health Network

Note that full information on the approval of the study protocol must also be provided in the manuscript.
